# Genomic Characterization of Epigenetic Regulator Gene Alterations in Juvenile Myelomonocytic Leukemia Through Whole-Exome Sequencing

**DOI:** 10.3390/epigenomes10030056

**Published:** 2026-08-19

**Authors:** Harsh Goel, Ravi Kumar Majhi, Jagdish Prasad Meena, Anita Chopra, Sameer Bakhshi, Lata Singh, Rachna Seth, Pranay Tanwar, Aditya Kumar Gupta

**Affiliations:** 1 Laboratory Oncology Unit, Dr. B.R.A. Institute of Rotary Cancer Hospital, All India Institute of Medical Sciences, New Delhi 110029, India; goel.harsh271@gmail.com (H.G.);; 2 Division of Pediatric Oncology, Department of Pediatrics, All India Institute of Medical Sciences, New Delhi 110029, India; majhiravi655@gmail.com (R.K.M.);; 3 Department of Medical Oncology, Dr. B.R.A. Institute of Rotary Cancer Hospital, All India Institute of Medical Sciences, New Delhi 110029, India

**Keywords:** juvenile myelomonocytic leukemia, whole-exome sequencing, epigenetic dysregulation, chromatin remodeling, ASXL1, EP300, mutational signatures, therapeutic targets

## Abstract

**Background/Objectives**: Juvenile myelomonocytic leukemia (JMML) is a rare, highly aggressive form of pediatric myelodysplastic/myeloproliferative neoplasm characterized by molecular heterogeneity and constitutive activation of the RAS signaling pathway. This study aimed to characterize the mutational landscape, driver genes, mutational signatures, functional pathways, and therapeutic potential of mutated epigenetic regulator genes in JMML. **Methods**: Tumor and matched buccal swab samples were collected from 35 JMML patients, and whole-exome sequencing was performed. Somatic variants were called with GATK-Mutect2 and annotated with ANNOVAR. maftools and OncodriveCLUST were used for mutational profiling, co-occurrence analysis, driver gene identification, and protein domain mapping. Drug–gene interactions were explored using DGIdb, mutational signatures for genes were characterized by MutationalPatterns, and functional enrichment analysis was performed by the clusterProfiler package. **Results**: A total of 28 variants were detected in epigenetic regulator genes, with missense mutations being the most common class of variants and a C>T nucleotide substitution pattern being the most frequent. EP300, SETD2, and DNMT3B were the genes most frequently altered, with 8.57% of cases each, followed by ASXL1, BCORL1, ATRX, KMT2A, and TET2 (5.71% each). Driver gene analysis identified ASXL1 as the top candidate driver gene, followed by BCORL1, EP300, and SETD2. Functional enrichment analysis revealed a high number of genes involved in chromatin organization, histone modification, transcriptional regulation, and oncogenic signaling pathways. The mutational signatures identified were SBS5-like, which are dominated by C>T and T>C transitions, indicating endogenous mutational processes. Analysis of drug–gene interactions revealed KMT2A, EP300, and ATRX as the most interconnected and potentially actionable therapeutic targets. **Conclusions**: This study provides a comprehensive characterization of epigenetic regulator gene alterations in JMML and highlights the importance of epigenetic dysregulation in disease pathogenesis.

## 1. Introduction

Juvenile myelomonocytic leukemia (JMML) is a rare and highly aggressive myelodysplastic/myeloproliferative neoplasm (MDS/MPN) that involves uncontrolled proliferation of monocytic and granulocytic precursor cells in the bone marrow [1]. The annual incidence of JMML is approximately 1.2 cases per million children per year [2,3]. JMML represents 1–3% of all childhood leukemias, according to the World Health Organization (WHO) and the International Consensus Classification (ICC) [4,5]. One of the distinguishing biological characteristics of JMML is spontaneous proliferation of myeloid progenitor cells resulting from hypersensitivity to granulocyte-macrophage colony-stimulating factor (GM-CSF), leading to aberrant activation of downstream signaling pathways, which subsequently results in uncontrolled myelopoiesis [6]. JMML is also linked with inherited predisposition syndromes, such as neurofibromatosis type 1 (NF1) and Noonan syndrome, further underlining a genetic aspect to disease development [7]. Hyperactivation of the RAS signaling pathway is a major driver of the molecular pathogenesis of JMML. Around 90% of JMML cases have mutations in one of five canonical driver genes: PTPN11 (protein tyrosine phosphatase non-receptor type 11), NRAS (neuroblastoma RAS viral oncogene homolog), KRAS (Kirsten rat sarcoma viral oncogene homolog), NF1 (neurofibromin 1), and CBL (Casitas B-lineage lymphoma) [8,9]. Nearly 35% of JMML cases have been shown to have chromosomal abnormalities, with the most common being monosomy 7 in about 25% of patients, and other abnormalities in about 10% [10]. Although molecular diagnosis and supportive care have developed over the years, allogeneic hematopoietic stem cell transplantation (HSCT) is still the only curative therapy for most patients. However, recurrence following transplant is a significant problem, with a 5-year relapse rate of approximately 35% [1,11]. In addition to the canonical mutations of the RAS pathway, recent genome studies have highlighted the role of secondary mutations in approximately 50% of JMML cases affecting transcription factors, chromatin-remodeling proteins, epigenetic regulators, spliceosome components, and the DNA methylation machinery [9,12,13]. These include mutations in SETBP1 (SET binding protein 1), which have been linked to poor clinical prognosis and aggressive disease biology [14,15]. Other recurrent secondary alterations include those in genes encoding components of the spliceosome (e.g., ZRSR2, zinc finger, CCCH domain-containing 2, RNA-binding motif and serine/arginine-rich 2), transcription factors (e.g., RUNX1, runt-related transcription factor 1, and GATA2, GATA-binding protein 2), and members of the JAK-STAT signaling pathway (e.g., JAK3, Janus kinase 3, and SH2B3, SH2B adaptor protein 3) [9,13,16]. These secondary mutations are often linked to disease progression, clonal evolution, and higher risk of relapse after HSCT. Genetic changes that impact epigenetic regulatory mechanisms have now become recognized as a significant component of JMML pathogenesis and are found in around 15% of patients. Most of these modifications are in genes that encode chromatin-modifying proteins, such as ASXL1 and EZH2 [9,16]. Mutations were also reported for DNMT3A but with a lower frequency compared to other myeloid malignancies [9,17]. Functional studies have shown that reduced PRC2 activity correlates with alterations in the trimethylation of histone H3 lysine 27 (H3K27me3) as well as with increased acetylation of H3K27, which suggests that impaired PRC2 activity may be central to the pathogenesis and progression of JMML [13]. It is now known that epigenetic dysregulation is a hallmark of JMML. Three distinct DNA methylation subgroups have been identified by genome-wide methylation studies: low methylation (LM), intermediate methylation (IM), and high methylation (HM), which strongly relate to the biology of the disease and prognosis. These results suggest that the DNA methylation profile could be used as a prognostic marker, as well as for molecular risk stratification in JMML [18,19,20]. Although there is growing evidence that epigenetic changes are involved in JMML pathogenesis, the detailed mutational profile and functional role of epigenetic regulator genes are only poorly understood. In the present study, we comprehensively characterize the mutational landscape of epigenetic regulator genes in JMML.

## 2. Results

### 2.1. Distribution and Classification of Somatic Variants in the Study Cohort

**Figure 1:**
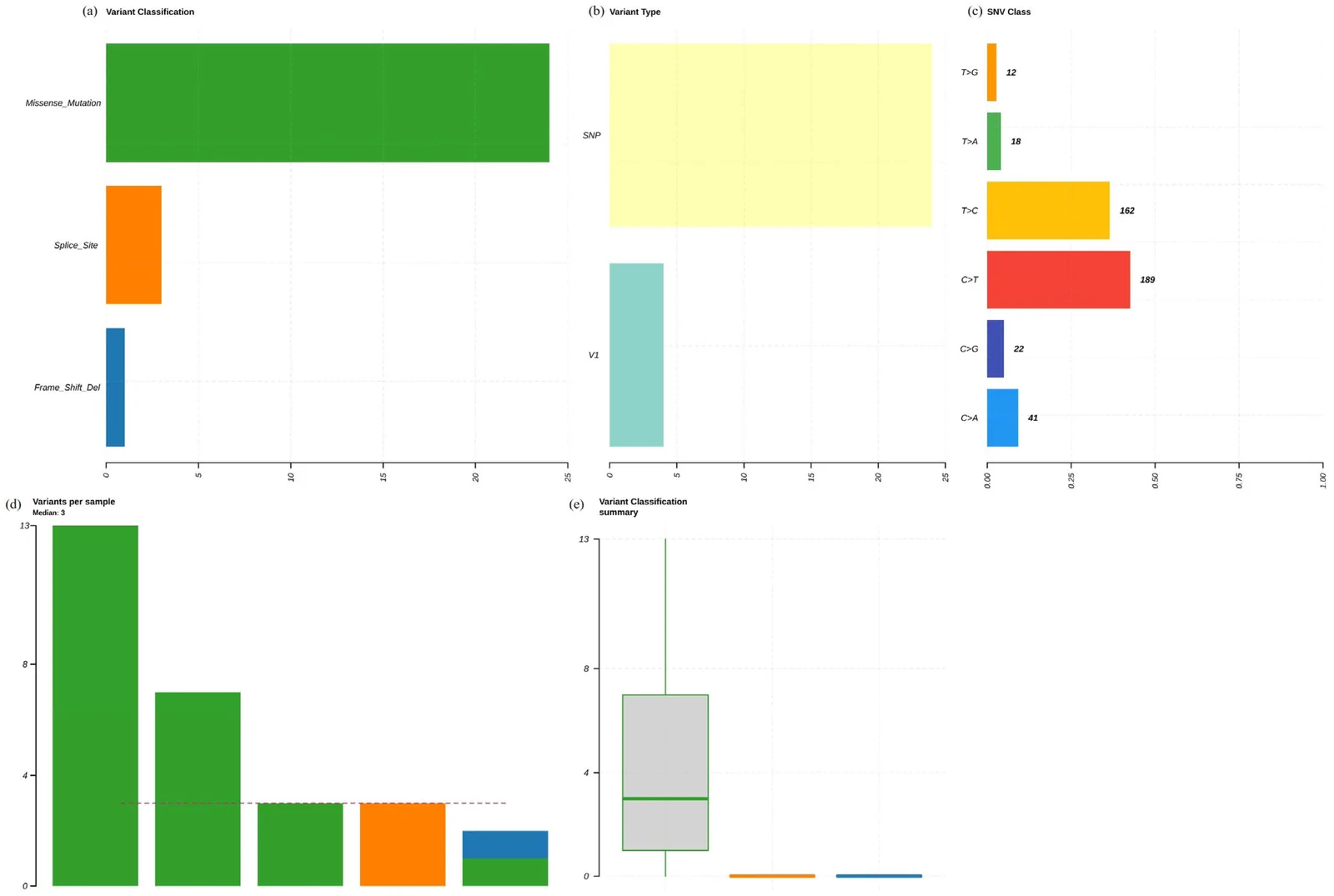
Characterization of somatic variants in the study cohort. (**a**) Variant classification distribution with the majority of missense mutations. (**b**) The distribution of variant types showing the SNPs as the predominant class of variants. (**c**) SNV classification profile showing enrichment of C>T substitutions. (**d**) Distribution of variants per sample; the dashed line shows the median number of mutations per sample. (**e**) Summary of variant classification by samples.

Somatic variants were analyzed according to variant classification, mutation type, single nucleotide variant (SNV) spectrum, mutation burden, and recurrently altered genes to characterize the mutational profile of the study cohort (Figure 1). A total of twenty-eight somatic variants in epigenetic regulator genes were identified in the analyzed samples. Missense mutations were also the most common type of alteration (24 mutations) among the variant classifications, suggesting that amino acid-altering substitutions were the most prevalent mutations in the cohort. In addition, splice-site mutations (n = 3) and frame-shift deletions (n = 1) were also observed at lower frequencies (Figure 1a). When the types of variants were assessed, the largest proportion of the variants was found to be single nucleotide polymorphisms (SNPs), with only a small proportion corresponding to insertion/deletion (indel) events (Figure 1b). This observation indicates that most of the mutations found in the analyzed samples were point mutations. The SNV spectrum was analyzed to determine the predominant type of substitution. The most common type of substitution was the C>T transition (n = 189), followed by the T>C transition (n = 162). Other substitution classes included C>A (n = 41), C>G (n = 22), T>A (n = 18), and T>G (n = 12) (Figure 1c), whereas the sample-level transition/transversion analysis presented in Figure 3 was performed to characterize inter-sample variation in substitution patterns and to quantify the relative contributions of transitions and transversions. Although 35 JMML patients were included in the overall whole-exome sequencing analysis, Figure 1d,e display five samples because these were the only patients harboring at least one mutation in the selected epigenetic regulator gene set used for the focused downstream analysis. The remaining 30 patients did not contain mutations in the selected genes and therefore did not generate variant records in the restricted MAF used for these visualizations. Thus, the five samples shown in Figure 1d,e were not selected as a representative subset, but correspond to the mutation-positive samples within the predefined epigenetic regulator gene set.

### 2.2. Recurrent Mutational Landscape of Epigenetic Regulator Genes in JMML

**Figure 2:**
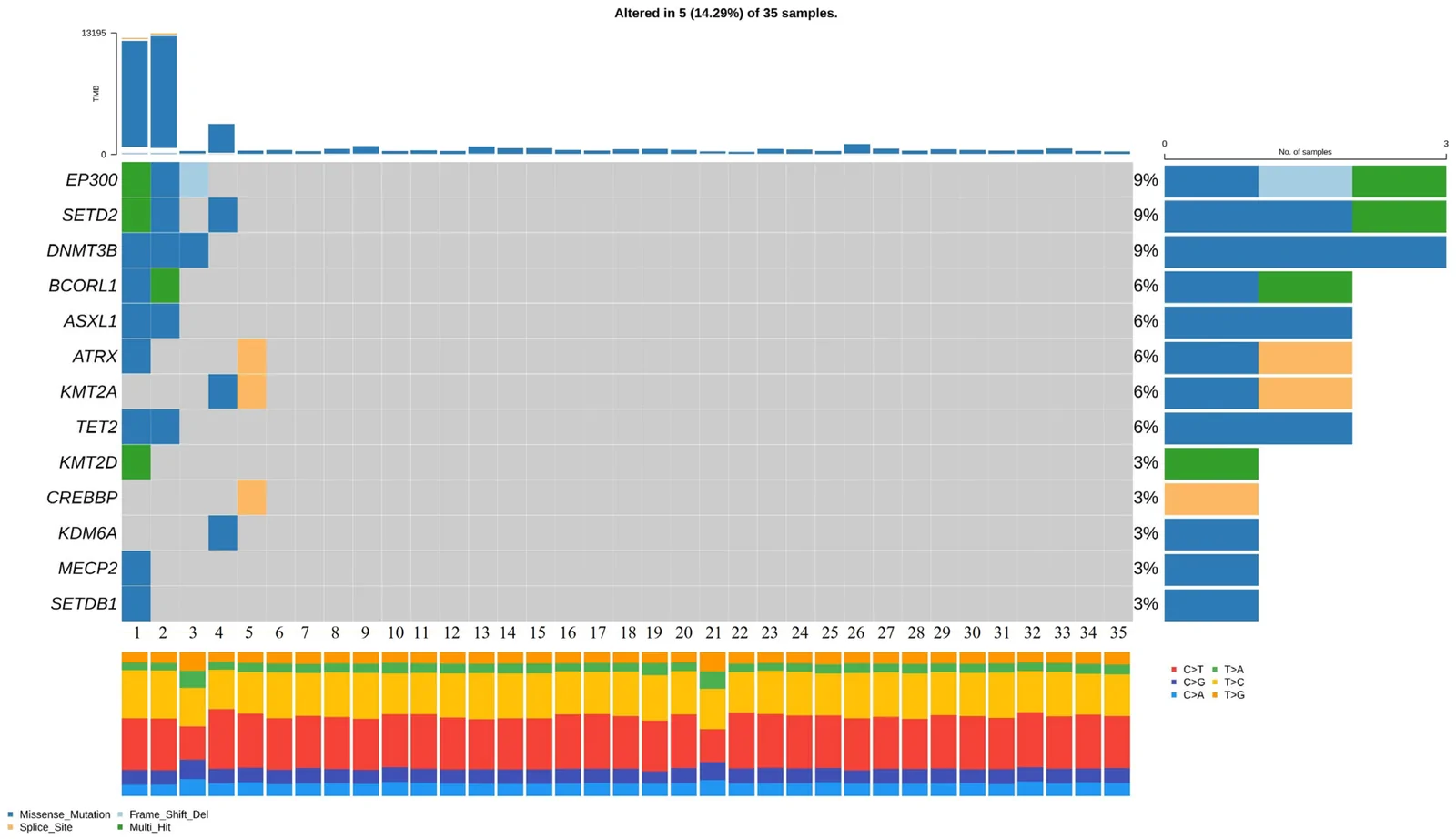
Recurrent mutational landscape and oncoplot representation of the study cohort. The oncoplot shows the distribution of recurrently mutated genes, along with mutation classes, tumor mutation burden (TMB), and SNV signatures in 35 samples. The upper graph shows the distribution of TMB within samples, and the middle graph shows the mutation pattern per gene. The bottom panel is a summary of SNV substitution classes.

To understand the genomic architecture of the cohort, an oncoplot analysis was performed to assess the distribution of recurrently mutated epigenetic genes, mutation type, tumor mutation burden (TMB), and single nucleotide variant (SNV) signatures across all the samples (Figure 2). Among the 35 analyzed samples, alterations in recurrently mutated genes were identified in 5 samples (14.29%), revealing a distinct mutational subgroup within the overall sample (Figure 2). The oncoplot revealed heterogeneous distribution patterns of somatic mutations among samples, with most alterations predominantly represented by missense mutations, whereas a limited number of splice-site variants, frame-shift deletions, and multi-hit events were also observed. Consistent with the findings from the overall mutational profiling, EP300, SETD2, and DNMT3B emerged as the most frequently altered genes, each mutated in 9% of samples. These alterations were primarily composed of missense mutations, although additional mutation classes including splice-site events and multi-hit alterations were also detected in selected samples. Other recurrently altered genes included BCORL1, ASXL1, ATRX, KMT2A, and TET2, each exhibiting mutation frequencies of 6%, while KMT2D, CREBBP, KDM6A, MECP2, and SETDB1 were altered in 3% of samples. This mutational spectrum also showed enrichment of C>T substitutions followed by T>C substitutions in the samples analyzed, which further confirms the higher prevalence of transition mutations in the cohort. Lower frequencies of C>A, C>G, T>A, and T>G substitutions were also detected (Figure 2, lower panel). The tumor mutation burden (TMB) analysis showed that there was a high degree of heterogeneity between samples. A small number of samples showed very high TMB, while the majority showed relatively low burdens, indicating the presence of a hypermutated subset and a low-mutational-load subset within the sample set (upper panel of Figure 2). Overall, the recurrent mutational landscape identified a significant number of epigenetic regulators and chromatin-remodeling genes that were frequently mutated, such as EP300, SETD2, DNMT3B, ASXL1, and KMT2 family members, suggesting they may play a role in disease pathogenesis and molecular heterogeneity.

### 2.3. Mutational Signature Distribution and Transition/Transversion Profile of Epigenetic Regulator Gene Alteration

**Figure 3:**
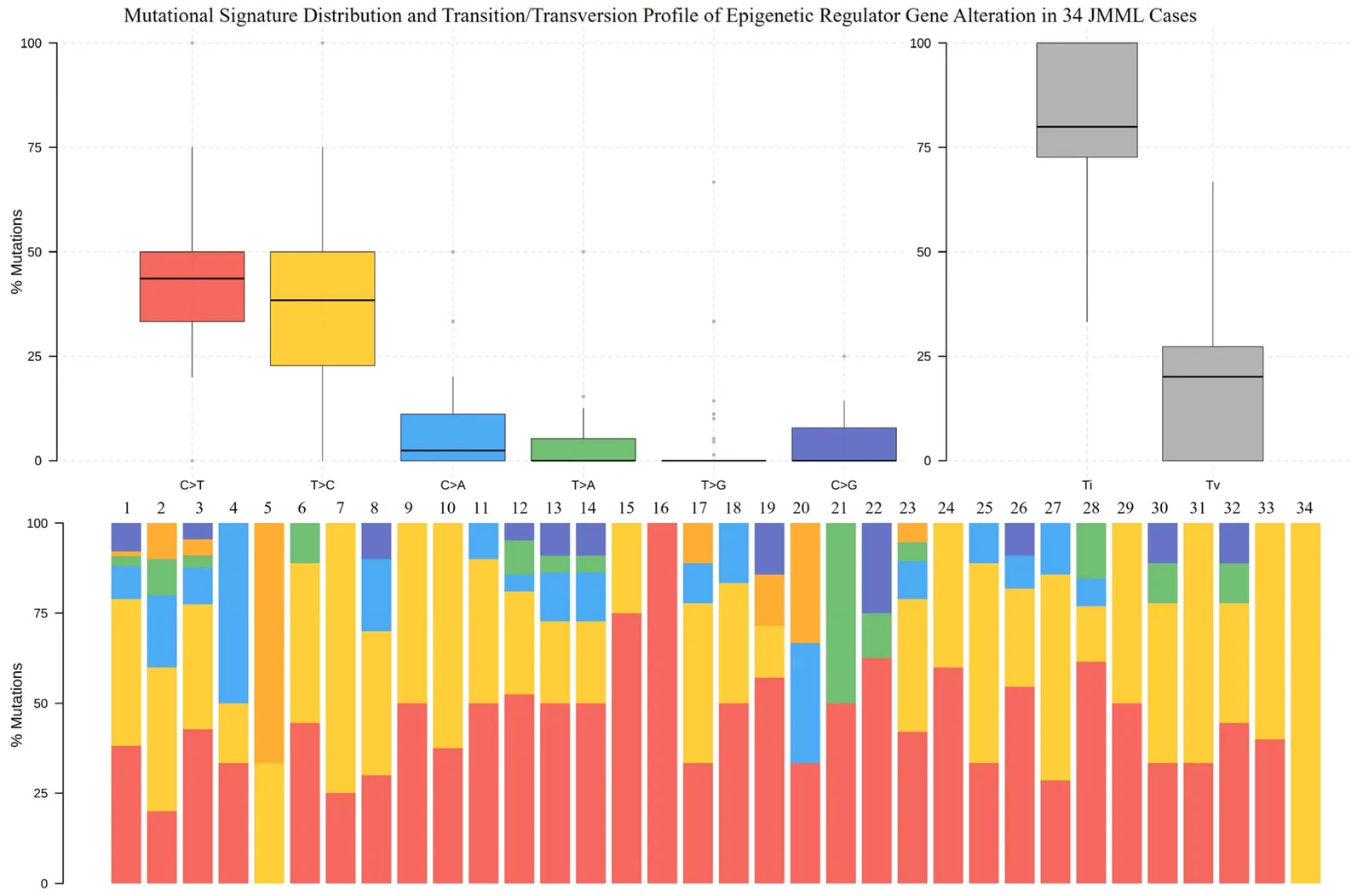
The mutational signature and transversion/transition pattern of the study cohort. (**a**) Boxplots showing the distribution of SNV substitution classes across samples. (**b**) Frequency distribution of the transition (Ti) and transversion (Tv) mutations. (**c**) Mutational signatures are shown as the sample-level composition and relative contribution of each mutation class (C>T, T>C, C>A, C>G, T>A, T>G) in the cohort.

The distribution of single-nucleotide variant (SNV) classes and transition/transversion (Ti/Tv) patterns was analyzed for somatic variants affecting the selected epigenetic regulator genes across the 35 JMML samples (Figure 3). Although 35 JMML samples were included in the Ti/Tv analysis, Figure 3 displays 34 samples because one sample contained no qualifying single-nucleotide variants within the predefined epigenetic regulator gene set. Consequently, this zero-variant sample was not displayed in the original maftools plotTiTv() visualization. Analysis of the SNV spectrum revealed that C>T substitutions were the most frequent mutation type found in our cohort, suggesting that they are the most common mutation type with a median frequency around 40–45% (Figure 3a). Similarly, T>C substitutions were prevalent as well, with median frequencies of about 35–40%, suggesting that transition mutations were the predominant type of mutation. Transversions were found to be less common. Of these, the median values of C>A substitutions were relatively low, with relatively small inter-sample variability, while T>A, T>G and C>G substitutions contributed a relatively small proportion of total mutations. Further assessment of transition (Ti) and transversion (Tv) events demonstrated a marked predominance of transition mutations, with transition frequencies reaching a median value of approximately 80%, whereas transversions accounted for a substantially lower proportion with median values around 20% (Figure 3b). This resulted in a high Ti/Tv ratio, supporting the dominance of transition-driven mutational processes within the cohort. Substantial variation was also observed among individual samples by the sample-wise mutational composition analysis (Figure 3c). The relative importance of each type of substitution was different in each sample, but the two most abundant categories were consistently the C>T and T>C substitutions, representing the major mutation categories, while C>A, C>G, T>A, and T>G substitutions contributed comparatively smaller proportions. Overall, the mutational signature analysis showed a mutational profile dominated by transitions, with the most prevalent ones being C>T and T>C substitutions, suggesting a shared mutational landscape among the samples and the presence of both common endogenous mutational processes and genomic heterogeneity.

### 2.4. Co-Occurrence and Mutual Exclusivity Analysis of Recurrently Mutated Genes

**Figure 4:**
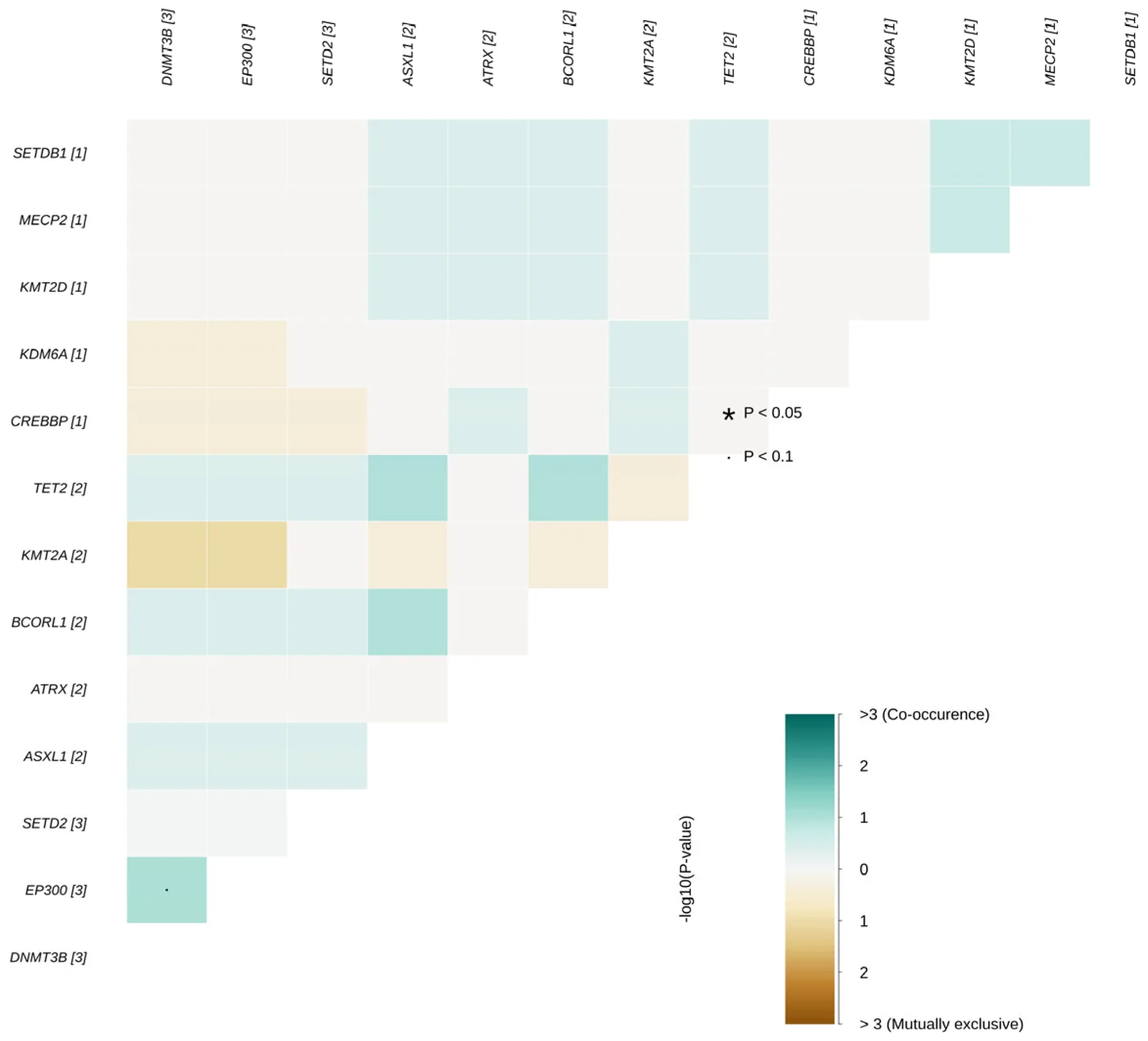
Co-occurrence and mutual exclusivity analysis of recurrently mutated genes. The heatmap shows the pairwise relationships between genes that are frequently mutated, as analyzed by co-occurrence and mutual exclusivity. Mutual co-occurrence of mutations is shown as positive values (green), and mutual exclusivity as negative values (brown). The strength of the color reflects the −log10(*p*-value). Significance is marked by symbols (* *p* < 0.05; *p* < 0.1).

Within the study cohort, co-occurrence and mutual exclusivity analyses were used to examine the relationships between recurrently mutated genes (Figure 4). A pattern of gene co-mutation and exclusivity relationships was identified that might indicate cooperative and alternative mutational events in the pathogenesis of the disease. The co-occurrence analysis revealed a tendency for several epigenetic regulator and chromatin-remodeling genes to occur together within the same samples. Notably, EP300 and DNMT3B demonstrated the strongest co-occurrence tendency, suggesting possible cooperative involvement in tumor development. Similarly, positive co-occurrence patterns were found between the genes TET2, BCORL1, ASXL1, ATRX, SETDB1, MECP2, and KMT2D, suggesting that these genes are often modified together. Among the recurrently altered genes, distinct co-occurrence patterns, e.g., BCORL1-ASXL1, TET2-ASXL1, and TET2-BCORL1 combinations, were highly noted for their co-occurrence patterns, which correspond to the coordinated involvement of genes associated with chromatin remodeling and epigenetic regulation. Additional positive associations were observed between CREBBP and ATRX, and between KDM6A and KMT2A, further supporting the role of transcription and histone modulations in the mutational network. There was a significant negative correlation between KMT2A and DNMT3B, KMT2A and EP300, and CREBBP and EP300 and SETD2; these correlations indicated possible functional redundancy or alternative ways to disrupt the pathways. Most associations were not statistically significant, though, given the limited number of altered samples, the observed co-occurrence and exclusivity patterns suggest an interconnected group of epigenetic and chromatin-remodeling networks within the cohort. Overall, the analysis showed that genes that were recurrently mutated had both cooperative and mutually exclusive relationships, suggesting complex molecular interactions involved in disease heterogeneity.

### 2.5. Mutational Landscape of Epigenetic Regulator Genes in JMML

**Figure 5:**
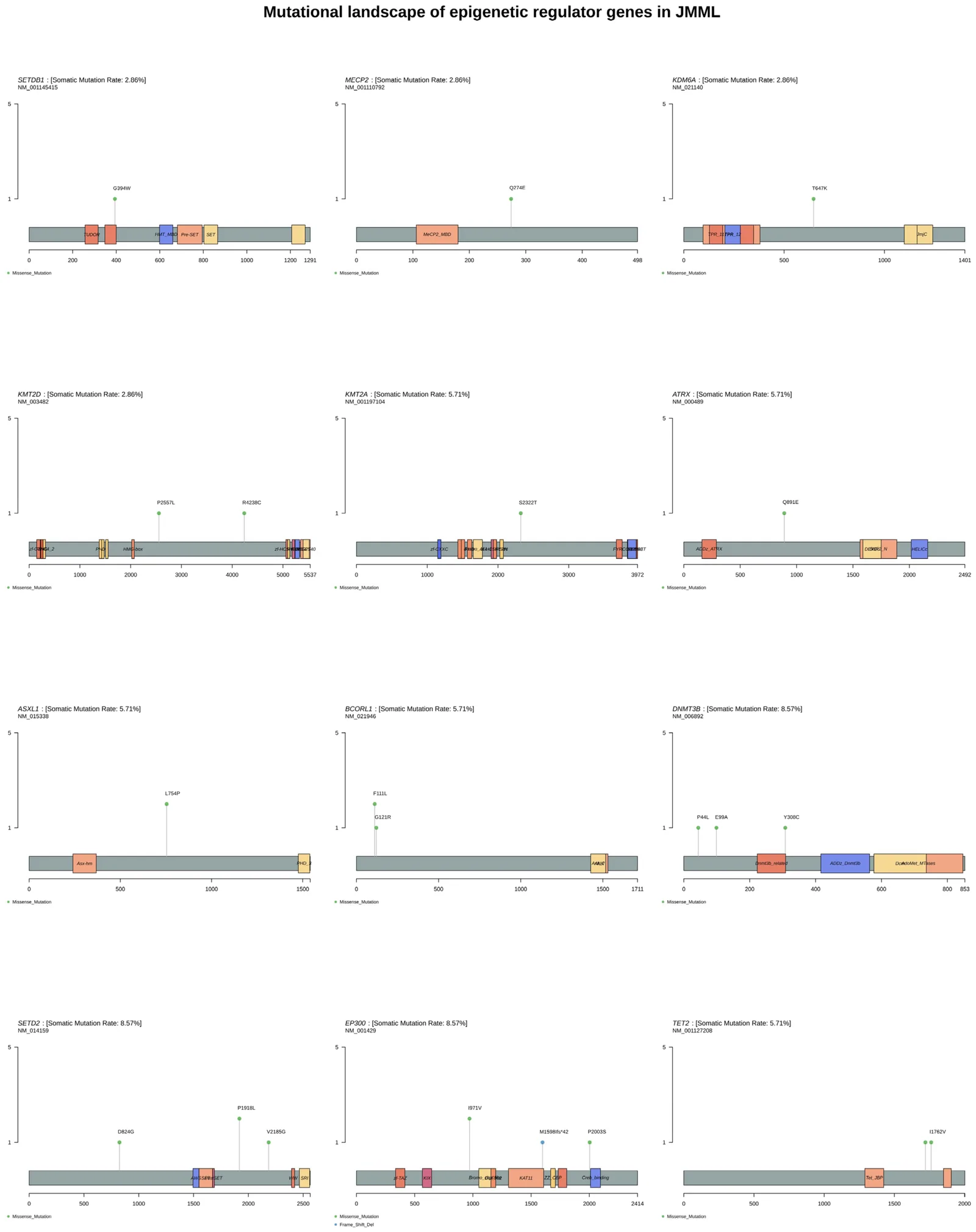
Protein domain-based mutational landscape of epigenetic regulator genes in JMML. Lollipop plots display the distribution of somatic mutations by protein domains in genes that are commonly mutated in epigenetic regulators.

To provide further characterization of the functional consequences of the recurrent somatic alterations, the mutational distribution of epigenetic regulator genes was analyzed at the protein domain level in JMML samples (Figure 5). The highest mutation frequencies were observed in DNMT3B (8.57%), SETD2 (8.57%), and EP300 (8.57%). Most of the mutations in these genes were missense mutations: P44L, E99A, and Y308C (DNMT3B), D824G, P1918L, and V2185G (SETD2), I971V and P2003S (EP300). Genes with intermediate mutation frequencies (5.71%) included TET2, KMT2A, ATRX, ASXL1, and BCORL1. Most of the mutations in these genes were missense mutations: I1762V (TET2), S2322T (KMT2A), I971V, and Q891E (ATRX), L754P (ASXL1), G121R and F111L (BCORL1). Additional low-frequency mutations (2.86%) were found in SETDB1, MECP2, KDM6A and KMT2D. Most of the mutations in these genes were single missense mutations: G394W (SETDB1), Q274E (MECP2), T647K (KDM6A), and P2557L and R4238C (KMT2D). Despite their lower frequencies, these genes are known regulators of histone modification and chromatin organization, indicating broader epigenetic dysregulation within the cohort. Overall, the protein domain mapping analysis showed that most of the mutations were found in DNA methylation, histone modification, and chromatin-remodeling pathways, indicating that these pathways are important in the pathogenesis of JMML.

### 2.6. Identification of Candidate Driver Genes in Epigenetic Regulatory Pathways

**Figure 6:**
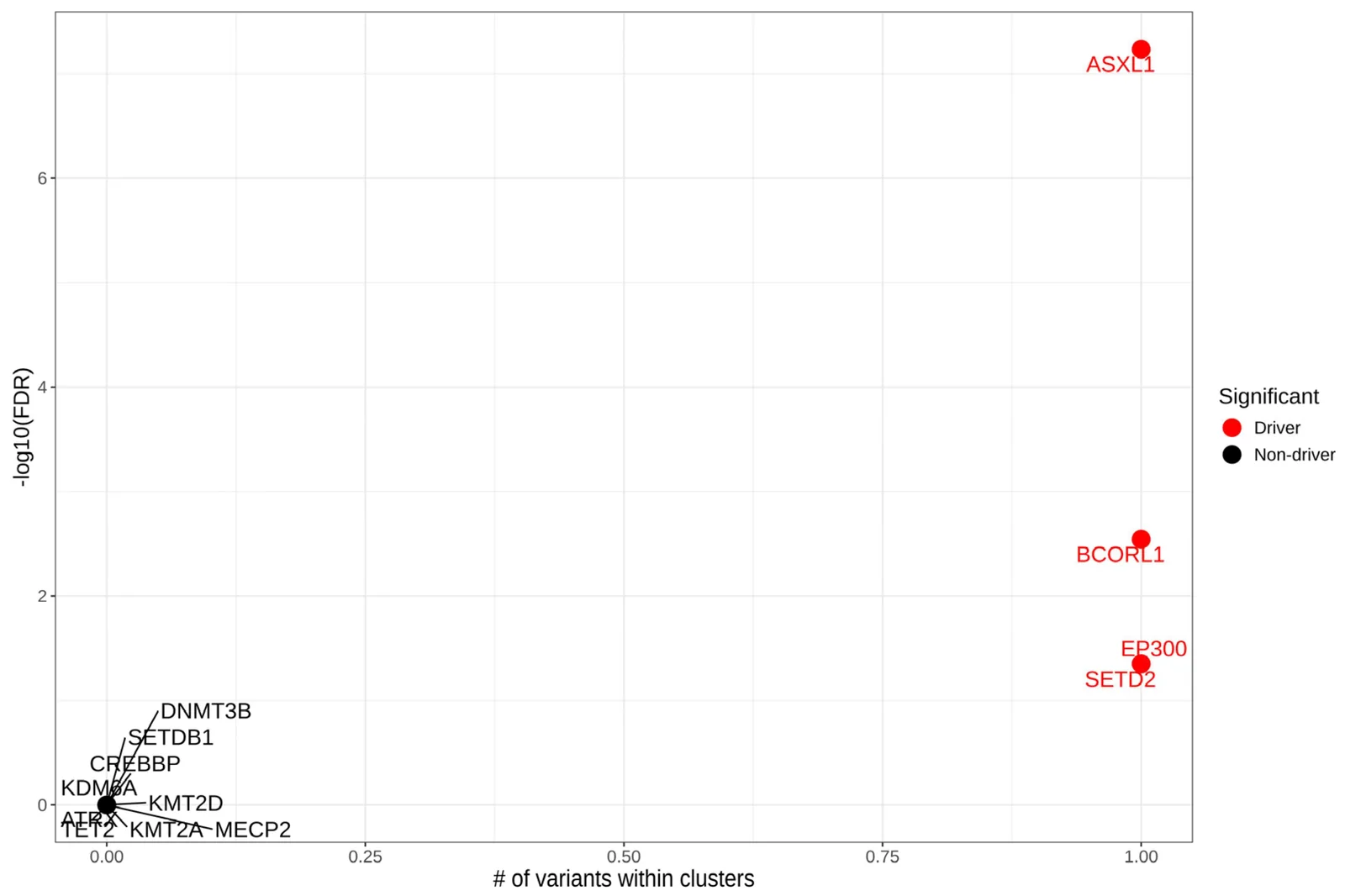
Driver gene analysis of recurrently mutated epigenetic regulator genes in JMML. The mutation clustering and statistical significance of recurrently altered genes are plotted as a scatter plot. Variants in clusters are plotted on the *x*-axis and significance on −log10(FDR) on the *y*-axis. Red dots represent candidate driver genes, and black dots represent non-driver genes.

Functionally relevant mutations that might be involved in the pathogenesis of JMML were analyzed by driver gene analysis using mutation clustering (Figure 6). Several genes were found to be repeatedly mutated and were significantly enriched for mutations, suggesting that they may be possible candidate driver events in the disease. Among all the genes analyzed, the gene ASXL1 was identified as the most important candidate driver gene due to its high statistical significance with an FDR value corresponding to −log10(FDR) > 7, which suggests strong evidence of positive selection and functional relevance in JMML (Figure 6). Moreover, ASXL1 showed mutation clustering, which further confirmed its role as an important pathogenic event. Another protein with significant enrichment was SETD2, which also showed a significant positive value and clustered mutations. Additionally, BCORL1 and EP300 were significantly enriched driver candidates with mutation clusters and significant positive values. BCORL1 demonstrated moderate significance (−log10(FDR) = 2.5), whereas EP300 and SETD2 showed lower but statistically relevant enrichment (−log10(FDR) = 1–1.5). The results indicate that changes in genes that are part of the epigenetic regulatory pathway and chromatin remodeling can play a role in the development of JMML. Genes such as DNMT3B, SETDB1, CREBBP, KDM6A, ATRX, TET2, KMT2A, KMT2D, and MECP2, however, had only marginal clustering and low significance values, suggesting that they may be non-driver mutations in our cohort. Although these genes were recurrently mutated, their lower enrichment suggests possible passenger roles or secondary contributions to disease progression. The overall driver analysis revealed ASXL1 as the primary candidate driver gene, along with the contribution of BCORL1, EP300, and SETD2, which all play a role in epigenetic dysregulation and chromatin-remodeling pathways and are consequently important in the pathogenesis of JMML.

### 2.7. Drug–Gene Interaction Network and Druggability Landscape of Epigenetic Regulator Genes in JMML

**Figure 7:**
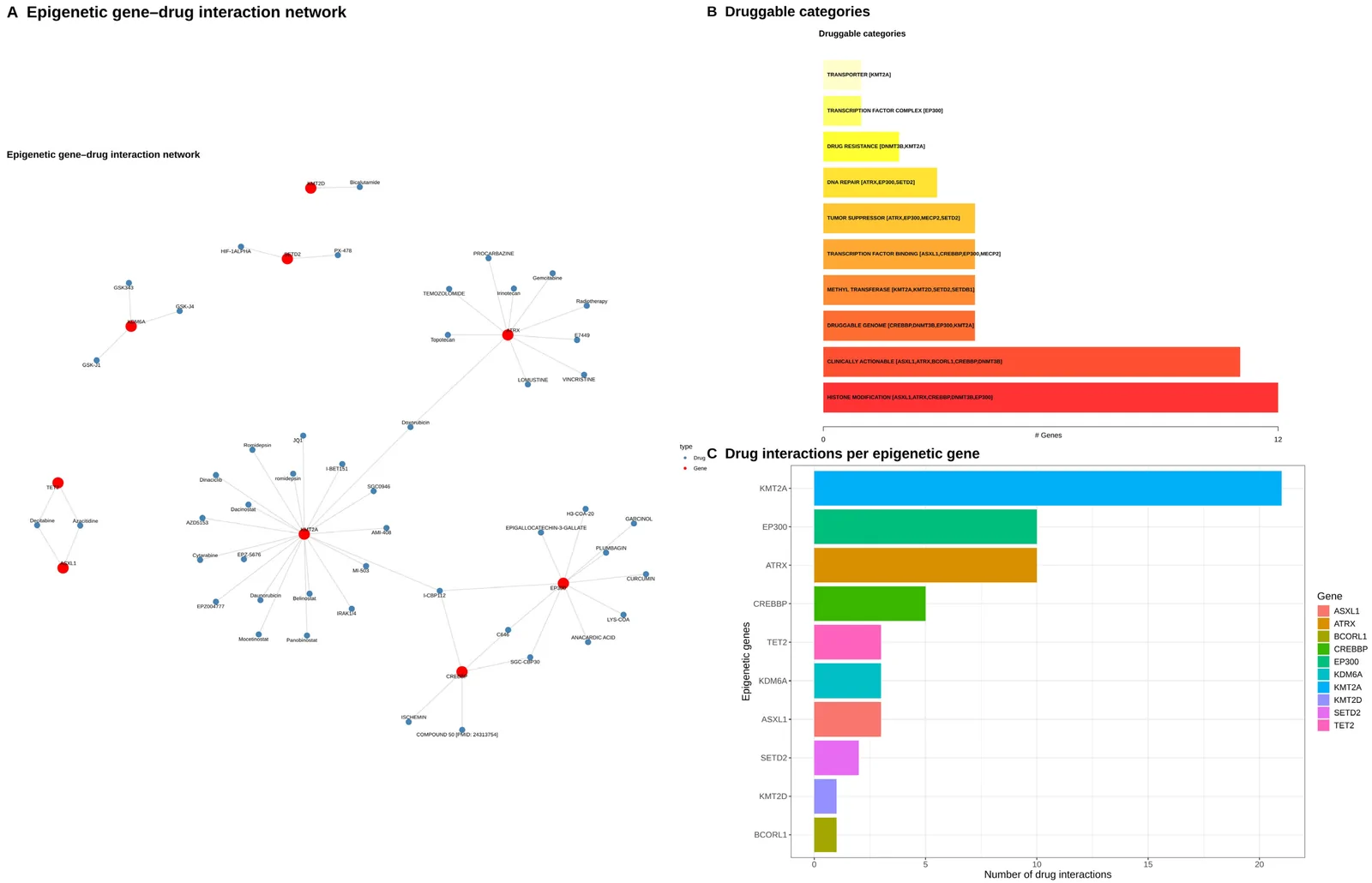
Drug–gene interaction and druggability landscape of epigenetic regulator genes in JMML. (**A**) Epigenetic gene–drug interaction network depicting the interaction between the recurrently mutated genes and therapeutic drugs. Genes are depicted by red nodes and interacting drugs are depicted by blue nodes. (**B**) Druggability classification of altered genes across functional categories including histone modification, clinically actionable targets, methyltransferases, DNA repair, and tumor suppressors. (**C**) Distribution of drug interactions per epigenetic gene.

Drug–gene interaction analysis and druggability assessment were performed on genes that were recurrently mutated in JMML to explore the therapeutic potential of these genes (Figure 7). The analysis revealed extensive interactions between epigenetic genes and candidate therapeutic compounds, highlighting several potentially actionable targets. The epigenetic gene–drug interaction network showed that KMT2A had the most drug interactions and was the most interconnected target in the network (Figure 7A,C). Multiple compounds were associated with KMT2A, including chemotherapeutic agents, kinase inhibitors, and epigenetic modulators, indicating broad therapeutic relevance. Other highly connected genes included EP300 and ATRX, each showing substantial interactions with multiple compounds. EP300 was found to interact with diverse epigenetic and natural compounds such as curcumin, plumbagin, anacardic acid, epigallocatechin-3-gallate (EGCG), and other modulators, reflecting its role as a potentially druggable transcriptional regulator. Similarly, ATRX interacted with numerous agents, including temozolomide, irinotecan, topotecan, vincristine, lomustine, and other anticancer agents, indicating potential therapeutic applications for chromatin-remodeling pathways. Moderate interaction profiles were observed for genes such as CREBBP, TET2, KDM6A, ASXL1, and SETD2, while the genes KMT2D and BCORL1 had comparatively fewer associated drugs (Figure 7C). Even though there were fewer interactions, these genes are still biologically relevant, as they are involved in epigenetic regulation. Druggability classification analysis further demonstrated that the altered genes were enriched within several therapeutically relevant categories (Figure 7B). Histone modification regulators were the largest group, highlighting the relevance of chromatin-modifying pathways in JMML, as were genes related to histone modification regulators such as ASXL1, ATRX, CREBBP, DNMT3B and EP300. Additional enriched categories comprised clinically actionable genes, druggable genome components, methyltransferases, transcription factor binding proteins, tumor suppressors and DNA repair genes. However, several genes were also identified as clinically actionable and druggable, including ASXL1, ATRX, BCORL1, CREBBP, DNMT3B, EP300, KMT2A, SETD2 and TET2, suggesting potential utility for targeted therapeutic development. Overall, the analysis of the drug–gene interaction network identified KMT2A, EP300 and ATRX as the most interconnected therapeutic targets and revealed that the genes encoding epigenetic regulators that are recurrently altered in JMML have significant druggability potential, especially in histone modification and chromatin-remodeling pathways.

### 2.8. Functional Enrichment Analysis of Recurrently Mutated Epigenetic Regulator Genes

**Figure 8:**
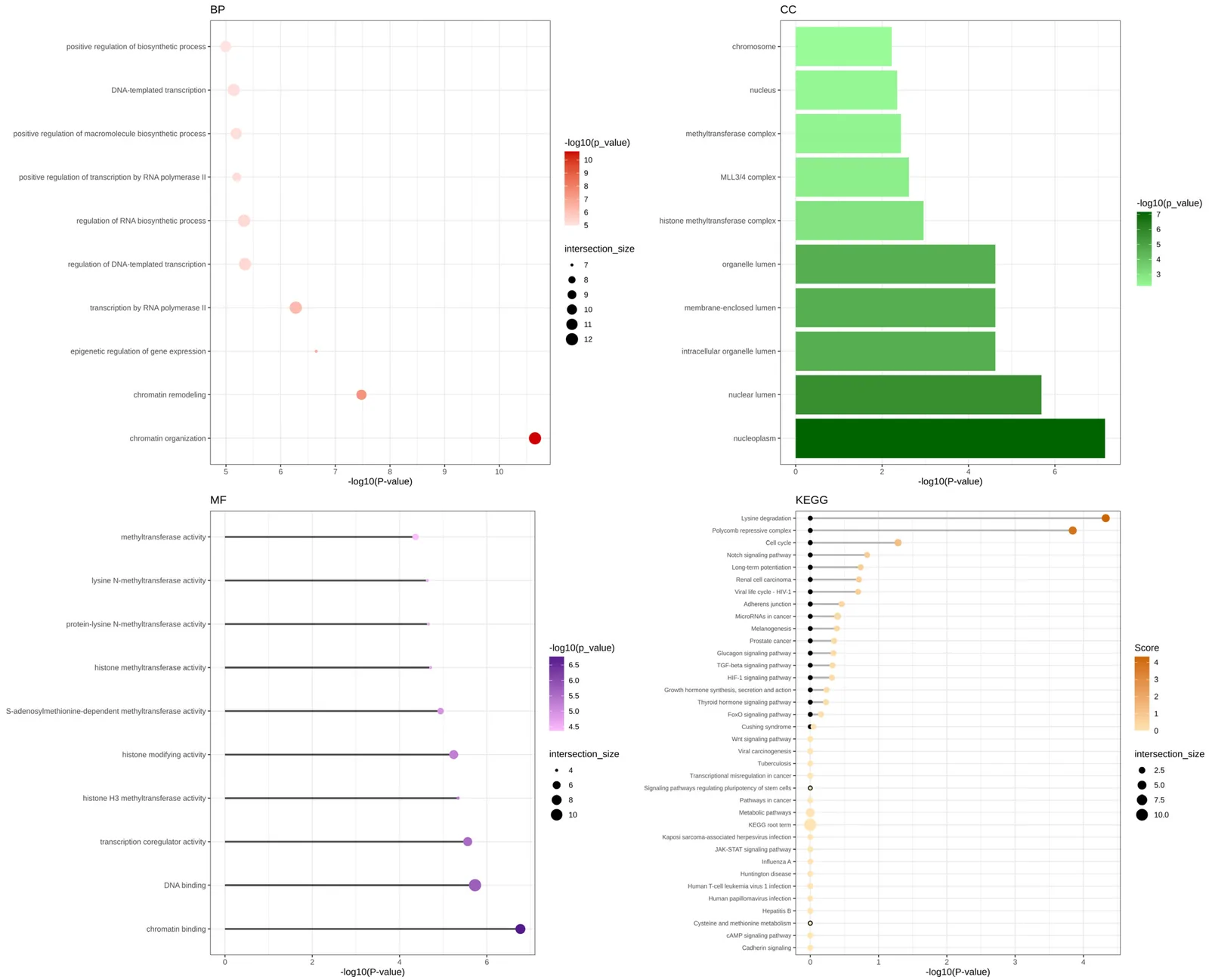
Functional enrichment analysis of recurrently mutated epigenetic regulator genes in JMML. (**a**) Gene Ontology biological process (BP) enrichment indicates significant enrichment in terms of chromatin organization, chromatin remodeling, and transcriptional regulation. (**b**) Cellular component (CC) enrichment demonstrating localization to nuclear and chromatin-associated compartments. (**c**) Molecular function (MF) enrichment highlighting chromatin binding, methyltransferase activity, and histone modification functions. (**d**) KEGG pathway enrichment reveals involvement of lysine degradation, Polycomb repressive complex pathways, cell cycle regulation, and major oncogenic signaling pathways including Notch, Wnt, TGF-β, and JAK–STAT signaling.

A functional enrichment analysis was conducted using Gene Ontology (GO) and Kyoto Encyclopedia of Genes and Genomes (KEGG) pathway annotations to investigate the biological significance of recurrently altered epigenetic regulators in JMML (Figure 8). The analysis showed significant enrichment of genes related to chromatin organization, transcription regulation, epigenetic modification, and oncogenic signaling pathways. Gene Ontology Biological Process (BP) analysis demonstrated that chromatin organization was the most significantly enriched biological process (−log10(*p*-value) > 10), followed by chromatin remodeling, indicating a major role of altered epigenetic regulators in maintaining chromatin architecture (Figure 8a). Additional enriched biological processes were epigenetic regulation of gene expression, transcription by RNA polymerase II, regulation of DNA-templated transcription, regulation of RNA biosynthetic processes, and positive regulation of macromolecule biosynthesis, further emphasizing transcriptional dysregulation in JMML. The Cellular Component (CC) analysis showed strong enrichment in nuclear-associated compartments (Figure 8b). Most significantly enriched components were nucleoplasm, nuclear lumen, intracellular organelle lumen, and membrane-enclosed lumen. Enrichment was also observed for histone methyltransferase complexes, MLL3/4 complexes, methyltransferase complexes, chromosomes, and the nucleus, which suggests that mutated genes are localized in chromatin-associated structures. Enrichment of activities involved in epigenetic regulation and chromatin interaction was also confirmed by Gene Ontology Molecular Function (MF) analysis (Figure 8c). The most highly enriched molecular functions were chromatin binding; transcription co-regulator activity; histone modifying activity; histone methyltransferase activity; protein-lysine N-methyltransferase activity; lysine N-methyltransferase activity; and S-adenosylmethionine-dependent methyltransferase activity. These findings indicate disruption of histone modification and methylation-associated mechanisms. To identify pathways related to cancer progression and epigenetic regulation, we performed KEGG pathway enrichment analysis (Figure 8d). The Lysine degradation pathway and the Polycomb repressive complex pathway were the two most significantly enriched pathways, indicating the role of histone modification processes. Additional enriched pathways comprised cell cycle regulation, Notch signaling, Wnt signaling, TGF-β signaling, HIF-1 signaling, FoxO signaling, and JAK–STAT signaling pathways. In addition, enrichment of other cancer-related pathways such as microRNAs in cancer, prostate cancer, renal cell carcinoma, and viral carcinogenesis was observed. Notably, the RAS signaling pathway was not significantly enriched among the recurrently mutated epigenetic regulator genes analyzed in this study. This finding reflects the predefined epigenetic regulator gene set used for enrichment analysis and should not be interpreted as evidence against the established role of RAS pathway activation in JMML. Overall, functional enrichment analysis showed that the key functional processes of epigenetic regulators that are frequently mutated are mainly related to chromatin organization, histone modification, transcription regulation, and oncogenic signaling pathways, indicating that epigenetic regulation is a key molecular pathway involved in JMML pathogenesis.

### 2.9. Mutational Signature Analysis of Epigenetic Regulator Genes in JMML

**Figure 9:**
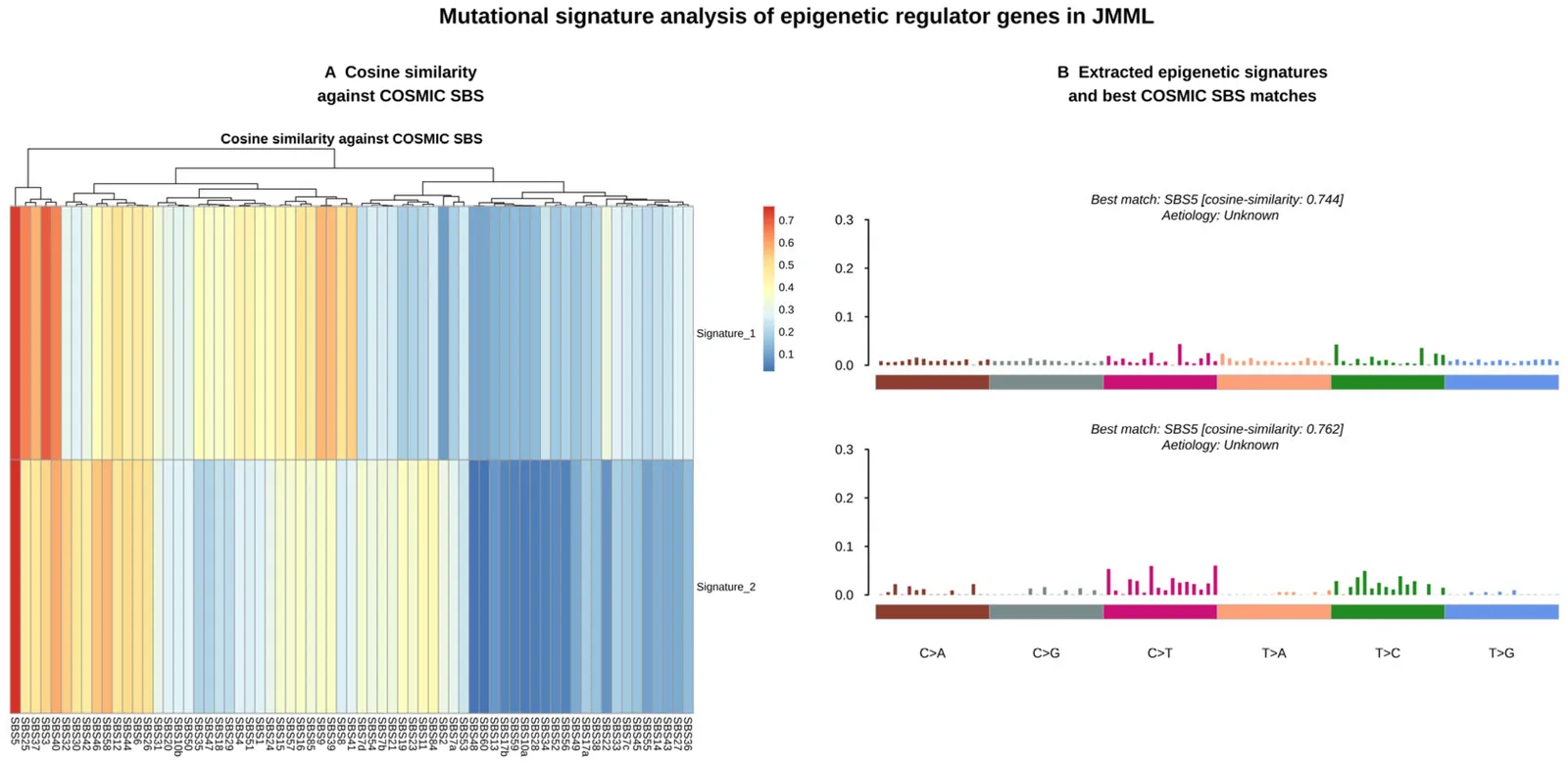
Mutational signature analysis of epigenetic regulator genes in JMML. (**A**) Heatmap indicating the cosine similarity between the extracted mutational signatures and COSMIC SBS reference signatures. (**B**) Extracted mutational signatures and their best COSMIC matches.

A mutational signature analysis was conducted to further characterize the mutational processes in JMML cells with altered epigenetic regulator genes by comparison with reference COSMIC single-base substitution (SBS) signatures (Figure 9). Two major mutational signatures with distinct substitution patterns and similarity profiles were identified for the analysis. JMML epigenetic signatures clustered together with COSMIC SBS5 when compared using cosine similarity analysis, showing that both signatures are likely to share a similar mutational process for epigenetic changes in JMML (Figure 9A). The cosine similarity values of Signature_1 and Signature_2 with SBS5 were moderate to high, indicating that the mutational process of epigenetic regulator genes shares characteristics with the SBS5 mutational process. Detailed comparison of extracted signatures revealed that Signature_1 showed the highest similarity with SBS5 with a cosine similarity score of 0.744, whereas Signature_2 demonstrated an even stronger association with SBS5 with a cosine similarity value of 0.762 (Figure 9B). SBS5 is classified as a clock-like mutational signature with unknown etiology and is frequently found in a wide range of cancers (according to COSMIC annotation). Both signatures were composed largely of C>T substitutions, with some T>C transitions, consistent with the previous analyses, which found the mutational spectrum to be dominated by transitions. Lower contributions from C>A, C>G, T>A, and T>G substitutions were also detected, indicating additional minor mutational processes. Notably, Signature_2 showed higher CT peaks of enrichment than Signature_1, indicating that different mutational processes associated with transitions are enriched in epigenetic regulator genes. The predominance of transition mutations further supports the involvement of endogenous mutational mechanisms in JMML pathogenesis. Overall, mutational signature analysis showed that signatures associated with recurrent alterations of epigenetic regulator genes were associated with COSMIC SBS5-like signatures, suggesting that mutational processes and mechanisms of endogenous genomic instability can play a role in epigenetic dysregulation in JMML.

## 3. Discussion

Juvenile myelomonocytic leukemia (JMML) is a genetically heterogeneous pediatric myelodysplastic/myeloproliferative neoplasm with the activation of the RAS signaling pathway in most cases [1,9]. Although significant progress has been made in elucidating the molecular mechanisms underlying JMML, the treatment of the majority of affected individuals is allogeneic hematopoietic stem cell transplantation (HSCT), and relapse still remains a significant clinical problem [1,11]. In the present study, we performed tumor-normal paired whole-exome sequencing to comprehensively characterize the mutational landscape of epigenetic regulator genes in JMML. Beyond the canonical RAS pathway mutations, our findings demonstrate recurrent alterations in genes involved in chromatin remodeling, histone modification, DNA methylation, and transcription regulation. Consistent with previous genomic studies, mutations of epigenetic regulator genes were found in about 14% of cases, which indicates that epigenetic dysregulation is involved in the pathogenesis of JMML in a biologically distinct subset of patients [9,13]. The most frequently altered genes were EP300 (8.57%), SETD2 (8.57%), and DNMT3B (8.57%), while ASXL1, BCORL1, ATRX, KMT2A, and TET2 were altered in 5.71% of patients. One of the genes that was most commonly changed in our cohort was EP300. EP300 is a histone acetyltransferase involved in chromatin accessibility, transcriptional activation, and cell differentiation. Mutations in EP300 have been found in various myeloid leukemias, but their significance has not been fully understood in JMML [21]. Mutations of EP300 have been reported to be secondary events in clonal evolution and may be linked to other epigenetic changes to drive the progression of disease rather than its initiation [22]. Similarly, SETD2 was recurrently mutated in our cohort. The histone H3K36 trimethyltransferase SETD2 has been shown to have critical functions in transcription regulation, DNA repair, and maintenance of genomic integrity [23,24]. Recent experimental research has shown that expression of SETD2 and levels of H3K36me3 are decreased in JMML models with RAS mutations and in KrasG12D-driven JMML, suggesting a role for SETD2 as a tumor suppressor in disease pathogenesis [25]. We also found a recurrent alteration in DNMT3B, a de novo DNA methyltransferase that is essential for establishing and maintaining DNA methylation patterns. DNMT3B mutations are rare in JMML, but increased DNMT3B expression has been linked to highly methylated subgroups of JMML and poor clinical outcomes [26,27]. Other recurrent changes included the ASXL1, BCORL1, ATRX, KMT2A and TET2 genes, which are all involved in important chromatin regulation and transcriptional control pathways. ASXL1 mutations have been linked to aggressive disease biology and poor prognosis, while TET2 mutations are linked to epigenetic homeostasis and DNA demethylation. Changes in the ATRX gene were previously reported as secondary genetic events in JMML and could play a role in disease heterogeneity by disrupting chromatin remodeling [28]. Similarly, BCORL1 and KMT2A are known epigenetic regulators of transcriptional repression and chromatin organization, respectively, and may thus play a role in the progression of JMML [29,30].

A key finding of our study was the discovery of high-order co-occurrence relationships between multiple epigenetic regulators. In particular, EP300 and DNMT3B showed the highest co-mutation tendency, and other positive associations were observed between ASXL1, BCORL1, TET2, ATRX, SETDB1 and KMT2D. Though none of the associations reached statistical significance due to the limited number of cases, the observations made here suggest that several epigenetic changes can play a concerted role in disease evolution rather than occurring as stand-alone genetic events. Cooperative interactions may have a collective effect on chromatin remodeling, transcriptional regulation, and leukemic transformation. Driver gene analysis also revealed ASXL1 as the most important recurrently altered epigenetic regulator driver gene, and BCORL1, EP300, and SETD2 as secondary driver genes. These results are consistent with previous reports showing that mutations in ASXL1 are linked to aggressive disease phenotypes and clonal evolution of JMML and other myeloid malignancies [31,32,33]. The discovery of further candidate driver genes (BCORL1, EP300, SETD2) indicates that chromatin-modifying pathways could be a major mechanism driving the pathogenesis and disease heterogeneity of JMML. These results were supported by functional enrichment analysis, which showed significant enrichment of pathways involved in chromatin organization, histone modification, transcriptional regulation, and various oncogenic signaling pathways, such as JAK–STAT, Wnt, Notch, TGF-β, HIF-1, and FoxO signaling pathways. These observations indicate that recurrently altered epigenetic regulators are involved in a variety of cellular processes that are central to the process of hematopoiesis, proliferation, and leukemic progression. Although RAS signaling is a central molecular pathway in JMML, the RAS signaling pathway was not significantly enriched in our focused analysis of recurrently mutated epigenetic regulator genes. This finding is likely attributable to the restricted gene set used for functional enrichment analysis and does not imply that RAS signaling is not involved in JMML pathogenesis. Canonical alterations in PTPN11, NRAS, KRAS, NF1, and CBL account for the major molecular mechanism of RAS pathway activation in JMML, whereas the present study was specifically designed to characterize alterations in epigenetic regulatory genes. Thus, our findings suggest that epigenetic alterations may represent an additional layer of molecular dysregulation rather than an alternative to the established RAS-driven biology of JMML.

Mutational signature analysis indicated that the mutation spectrum was enriched in C>T mutations and that the most prominent mutation signatures were closely related to COSMIC SBS5, which is a clock-like signature linked to endogenous mutations [34,35]. Overall, the distribution of transition mutations, especially C>T and T>C substitutions, implies that intrinsic mutational mechanisms play an important role in the occurrence of somatic mutations in JMML. While the biological role of SBS5 in JMML is not completely understood, its presence is compatible with endogenous genomic processes playing a role in disease evolution and epigenetic dysregulation [19,20]. However, the biological interpretation of individual substitution classes remains complex, and the present data cannot establish a specific causative mutational mechanism. Therefore, these findings should be considered as evidence of the mutational pattern associated with epigenetic regulator gene alterations rather than definitive evidence of a particular endogenous mutational process. From a translational perspective, the analysis of drug–gene interactions revealed KMT2A, EP300, and ATRX as the most connected therapeutic targets. Recently, a menin inhibitor, revumenib, has proven to be successful in treating relapsed or refractory KMT2A-rearranged leukemia, showcasing the therapeutic promise of chromatin-associated pathways [36,37]. EP300 is also a promising therapeutic target due to its interactions with multiple histone acetyltransferase inhibitors and the development of novel, selective EP300/CREBBP inhibitors, which have shown anti-leukemic properties in in vitro studies [38]. Moreover, genes that had been recurrently altered were enriched within clinically actionable groups, such as those involved in histone modification, methyltransferases, transcription factors, tumor suppressors, and DNA repair genes, highlighting the clinical importance of epigenetic dysregulation in JMML [23,31,39]. Epigenetic drugs, including hypomethylating agents (e.g., azacitidine), have already shown clinical activity in JMML, and other epigenetic therapies (e.g., EZH2 inhibitors) are emerging that could provide further opportunities for precision therapies in molecularly defined patient subsets [1,40].

There are several limitations to be taken into account when interpreting our findings. First, the small cohort size (n = 35), which is typical for a very rare disease such as JMML, limits the statistical power of the study and may influence the accuracy of mutation frequency estimates. Second, there were no comprehensive clinical outcome data, limiting our ability to assess prognostic associations between specific epigenetic alterations and clinical endpoints. Third, functional validation studies were not conducted; therefore, the biological and mechanistic significance of some of the novel or infrequently observed variants remains to be established. Although computational approaches, including driver-gene analysis, protein-domain mapping, functional enrichment, and drug–gene interaction analysis, provided evidence supporting the potential relevance of recurrently altered epigenetic regulator genes, these analyses cannot substitute for experimental validation. Fourth, the absence of complementary transcriptomic, methylation, and single-cell genomic datasets prevented us from defining detailed downstream molecular consequences of the mutations identified. Lastly, even though the study was conducted within one population cohort, the validity of the findings needs to be confirmed in larger multicenter and ethnically diverse populations to establish wider generalizability of the findings. In summary, our study not only provides a comprehensive characterization of epigenetic regulator gene mutations in JMML, but also highlights that recurrent mutations of chromatin remodeling, histone modification, and DNA methylation pathways are important to disease heterogeneity. The discovery of recurrent mutations in EP300, SETD2, DNMT3B, ASXL1, BCORL1, ATRX, KMT2A and TET2, and the identification of ASXL1 as a key driver gene and of therapeutically relevant epigenetic targets both reveal the biological and clinical relevance of epigenetic deregulation in JMML. These results contribute to our knowledge of the pathogenesis of JMML and could serve as a basis for future functional studies and epigenetic-based therapeutic approaches.

## 4. Materials and Methods

### 4.1. Patient Sample Collection and Whole-Exome Sequencing

Bone marrow/peripheral blood samples were collected with matched buccal swab samples from 35 patients with juvenile myelomonocytic leukemia (JMML) with informed consent of the parents/legal guardians. The study was conducted in accordance with the Declaration of Helsinki and was approved by the Institutional Ethics Committee of the All-India Institute of Medical Sciences (AIIMS), New Delhi, India (Approval No. IEC-351/08.5.2020, RP-41/2020). BM/PB samples were used for the isolation of mononuclear cells using the Ficoll–Paque density gradient centrifugation method. Tumor DNA and matched buccal swab DNA were extracted using the DNeasy Blood and Tissue Kit (Qiagen, Hilden, Germany) according to the manufacturer’s instructions. DNA concentration was quantified using Qubit fluorometry (Thermo Fisher Scientific, Waltham, MA, USA), and DNA integrity and purity were evaluated by the Agilent TapeStation 4200 system (Agilent Technologies, Santa Clara, CA, USA). Samples with acceptable DNA quality and A260/280 ratios within the range of 1.8–2.0 were selected for library preparation. Whole-exome libraries were prepared using the Agilent SureSelect Human All Exon V7 kit (Agilent Technologies, Santa Clara, CA, USA) following the manufacturer’s guidelines. Sequencing was performed on the Illumina NovaSeq 6000 sequencing platform (Illumina, San Diego, CA, USA) with paired-end sequencing chemistry (2 × 150 bp).

### 4.2. Somatic Variant Calling and Annotation

Raw sequencing reads obtained from whole-exome sequencing were assessed for quality using FastQC (v0.12.1). Adapter trimming and removal of poor-quality sequences were carried out with Trimmomatic (v0.39). The high-quality reads were mapped to the human reference sequence (hg19) with BWA-MEM (v0.7.17). Post-alignment processing of the reads was done according to the best-practice guidelines from the Genome Analysis Toolkit (GATK v4.4). Matched buccal swab samples were used from the same JMML patients as germline controls to identify somatic mutations from germline variants. Somatic single nucleotide variants (SNVs) and small insertions/deletions were detected across the exome in paired tumor-normal samples using Mutect2 (GATK) and filtered with FilterMutectCalls. High-confidence somatic alterations were retained by excluding variants that were detected in matched buccal swab samples. Variant annotation was done by ANNOVAR (version 2020-06-08) based on RefSeq, COSMIC (v96), and ClinVar databases. Common variants were further filtered out from population databases such as gnomAD, 1000 Genomes, and ExAC. The Mutation Annotation Format (MAF) files containing the exome-wide somatic variants were generated and processed in R (v4.3.1) using the maftools package.

### 4.3. Identification and Characterization of Epigenetic Regulator Genes

Following exome-wide somatic variant identification, genes involved in epigenetic regulation, chromatin remodeling, histone modification, DNA methylation, and transcriptional regulation were selected based on published literature and functional annotation databases for focused downstream analysis. Using the maftools package, mutational frequencies, variant types, mutation burden, and recurrent mutation patterns of epigenetic regulator genes were studied. Oncoplots were created to visualize the pattern of mutations across samples. Nucleotide substitution patterns were identified by transition and transversion (Ti/Tv) analysis, and mutational heterogeneity among samples was assessed.

### 4.4. Co-Occurrence, Mutual Exclusivity, and Driver Gene Analysis

Mutual exclusivity and co-occurrence analyses were conducted to explore interactions between recurrently altered genes in the epigenetic regulators. Pairwise interaction analysis was carried out with the somaticInteractions function from maftools. The statistical significance of mutational associations was tested by Fisher’s exact test, and interaction strengths were shown as −log10(*p*-values). Positive associations that were associated with each other were considered co-occurring mutations, with negative associations considered mutually exclusive mutations. OncodriveCLUST was used for driver gene analysis, which allows the identification of genes with accumulated mutations in certain protein domains. Statistical significance of mutation clustering was assessed using the OncodriveCLUST statistical framework, and *p*-values were corrected for multiple testing using the Benjamini–Hochberg method to obtain false discovery rate (FDR) values. Genes demonstrating significant mutation clustering were considered candidate driver genes.

### 4.5. Protein Domain Mapping and Mutation Visualization

The distribution of recurrent mutations in epigenetic regulator genes and their architecture in terms of protein domains were visualized with the lollipopPlot function from maftools. The protein domain information was derived from the annotation of protein transcripts and amino acid coordinates. The sequences of mutations in SETDB1, MECP2, KDM6A, KMT2D, KMT2A, ATRX, ASXL1, BCORL1, DNMT3B, SETD2, EP300, and TET2 were mapped onto the protein structures to assess recurrent mutation hotspots and functionally important domains.

### 4.6. Functional Enrichment and Pathway Analysis of Recurrently Mutated Epigenetic Regulator Genes

To identify the biological functions associated with the genes that were altered multiple times in the epigenetic regulators, we performed functional enrichment analysis using the clusterProfiler package implemented in R, and gene ontology (GO) enrichment analysis was conducted focusing on three categories: Biological Process (BP), Molecular Function (MF), and Cellular Component (CC). KEGG pathway enrichment analysis was also conducted to determine which signaling pathways were significantly enriched. Benjamini-Hochberg multiple testing correction was used, and pathways with adjusted *p*-values (FDR) < 0.05 were statistically significant.

### 4.7. Mutational Signature Analysis

Mutational signature analysis was performed with MutationalPatterns (v3.18) to characterize mutational processes associated with epigenetic regulator genes. Mutational signatures were extracted from somatic SNVs derived from MAF files by non-negative matrix factorization (NMF). The extracted signatures were compared to COSMIC mutational signatures (v3.3) via cosine similarity analysis. Cosine similarity heatmaps were used to visualize the similarities between signatures and best-matching COSMIC SBS signatures.

### 4.8. Drug–Gene Interaction and Therapeutic Target Analysis

The drug–gene Interaction Database (DGIdb v4.3.0) was used to investigate potential therapeutic targets related to the recurrently mutated epigenetic regulator genes. Drug interactions (both known and predicted) were screened to identify pharmacologically relevant therapeutic associations. igraph (v2.0.3) and Cytoscape (v3.10.2) were used to build and visualize drug–gene interaction networks. Genes were grouped by their functional annotations such as histone modifiers, methyltransferases, transcriptional regulators, tumor suppressors, DNA repair genes, and clinically actionable targets. Only curated and therapeutically relevant drug–gene interactions were retained for downstream analyses.

### 4.9. Statistical Analysis

The statistical analysis and graphical visualization were carried out using R software (v4.3.1) and Python (v3.2). Median and range values were used to summarize the values for continuous variables. Multiple testing correction was obtained using the Benjamini–Hochberg correction method, and adjusted *p*-values < 0.05 were considered statistically significant throughout the study.

## 5. Conclusions

Our results show that recurrent changes in EP300, SETD2, DNMT3B, ASXL1, BCORL1, ATRX, KMT2A and TET2 underscore the molecular diversity of JMML and the role of epigenetic dysregulation in the pathogenesis of the disease. The driver gene analysis identified ASXL1, BCORL1, EP300, and SETD2 as potential functional drivers of leukemogenesis, and functional enrichment analysis showed significant enrichment of chromatin organization, histone modification, transcriptional regulation, and oncogenic signaling pathways. The mutational signature analysis revealed an enrichment of transition mutations and the presence of SBS5-like signatures, indicating that endogenous mutational processes play a role in the generation of epigenetic changes in JMML. Moreover, drug–gene interaction analysis revealed that KMT2A, EP300, and ATRX are therapeutic targets potentially suitable for epigenetic-based treatment strategies. These results need to be confirmed in larger independent cohorts and in functional studies, but they contribute to the current knowledge of JMML biology and will serve as a basis for further precision medicine approaches and the discovery of new epigenetically targeted therapies.

## Data Availability

The data supporting the findings of this study are not publicly available due to ethical and privacy restrictions but are available from the corresponding author upon reasonable request.

## Abbreviations

The following abbreviations are used in this manuscript:

JMML	Juvenile myelomonocytic leukemia
PTPN11	Protein tyrosine phosphatase non-receptor type 11
NF1	Neurofibromatosis type 1
KRAS	Kirsten rat sarcoma
NRAS	Neuroblastoma rat sarcoma
CBL	Casitas B-lineage lymphoma
GM-CSF	Granulocyte-macrophage colony-stimulating factor
SETBP1	SET binding protein 1
JAK3	Janus kinase-3
ASXL1	Additional Sex Combs Like 1
RAS	Rat sarcoma
MAPK	Mitogen-Activated Protein Kinase
HLA	Human Leukocyte Antigen
WHO	World Health Organization
EP300	E1A Binding Protein P300
SETD2	SET Domain Containing 2
DNMT3B	DNA Methyltransferase 3 Beta
BCORL1	BCL6 Corepressor Like 1
ATRX	Alpha Thalassemia/Mental Retardation Syndrome X-Linked
